# Uncovering a Key Role of ETS1 on Vascular Abnormality in Glioblastoma

**DOI:** 10.3389/pore.2021.1609997

**Published:** 2021-11-19

**Authors:** Jiefu Tang, Yaling Li, Boxuan Liu, Wei Liang, Sanbao Hu, Meilian Shi, Jie Zeng, Mingzhen Li, Minjiang Huang

**Affiliations:** ^1^ Trauma Center, The First Affiliated Hospital of Hunan University of Medicine, Huaihua, China; ^2^ Department of Obstetrics and Gynaecology, Xi’an People’s Hospital (Xi’an Fourth Hospital), Xi’an, China; ^3^ Precision Medicine Center, The Second People’s Hospital of Huaihua, Huaihua, China; ^4^ Department of Orthopaedics, The Second People’s Hospital of Huaihua, Huaihua, China; ^5^ Department of Orthopaedics, Beijing Anzhen Hospital, Capital Medical University, Beijing, China; ^6^ Department of Infectious Diseases, The Second People’s Hospital of Huaihua, Huaihua, China; ^7^ Hunan University of Medicine, Huaihua, China

**Keywords:** glioblastoma, ETS1, endothelial, tumor vessel, vascular abnormality

## Abstract

Glioblastoma (GBM) is the most aggressive type of brain tumor. Microvascular proliferation and abnormal vasculature are the hallmarks of the GBM, aggravating disease progression and increasing patient morbidity. Here, we uncovered a key role of ETS1 on vascular abnormality in glioblastoma. ETS1 was upregulated in endothelial cells from human tumors compared to endothelial cells from paired control brain tissue. Knockdown of Ets1 in mouse brain endothelial cells inhibited cell migration and proliferation, and suppressed expression of genes associated with vascular abnormality in GBM. ETS1 upregulation in tumor ECs was dependent on TGFβ signaling, and targeting TGFβ signaling by inhibitor decreased tumor angiogenesis and vascular abnormality in CT-2A glioma model. Our results identified ETS1 as a key factor regulating tumor angiogenesis, and suggested that TGFβ inhibition may suppress the vascular abnormality driven by ETS1.

## Introduction

Glioblastoma (GBM) is the most aggressive and common primary malignant brain tumor with a dismal prognosis (1). Microvascular proliferation and abnormal vasculature are the hallmarks of GBM (2,3). Endothelial cells (ECs) in GBM vessels are associated with a distinct gene signature characterized by upregulation of genes involved in basement membrane remodelling, cytoskeletal rearrangements, angiogenic sprouting and tip cell formation (4). Vascular abnormality aggravates GBM progression by promoting tumor cell invasiveness through inducing hypoxia (5,6). In addition, GBM vessels contribute to tumor relapse by providing specialized niches for glioma stem-like cells (GSCs) (7). Moreover, GBM vessels are leaky and hyper-permeable, resulting in life-threatening edema (8). Therefore, vasculature in GBM has been identified as an attractive therapeutic target for treatment (7). Several signal pathways driving vascular abnormality in GBM have been uncovered, including hypoxia, transforming growth factor β (TGFβ), pleiotrophin (PTN) and vascular endothelial growth factor (VEGF) signaling pathways (9–12). Bevacizumab, a humanized anti-vascular endothelial growth factor (anti-VEGF) neutralizing antibody, has been approved for recurrent GBM by FDA but has not led to improved overall survival (13). Further knowledges revealing molecular mechanisms of vascular abnormality in GBM may improve the efficiency of current vessel-targeting therapies and yield novel therapeutic strategies (14).

ETS proto-oncogene 1, transcription factor (ETS1) belongs to the E26 transformation-specific (ETS) transcription factors family (15). ETS1 is highly expressed in immune cells and ECs, and its role on mediating T and B cell differentiation has been well characterized (16,17). Ets1 knockout in mice leads to aberrant T cell linage differentiation, characterized by decreased number of Th1, Th2 and Treg cells and defects in CD8 T cell development and function (18). In B cell linage, enhanced differentiation into IgM- and IgG-secreting plasma cells has been observed in Ets1 knockout mice (16). ETS1 is expressed at very low level in resting endothelium, but is transiently induced in ECs during angiogenesis and injury (19,20). Studies using cultured endothelial cells conclusively demonstrated the effects of ETS1 on angiogenesis and cell apoptosis *in vitro* (21–24). Mice lacking either Ets1 or Ets2 did not exhibit any apparent vascular abnormalities, but simultaneous knockout of ETS1 and ETS2 in mice leads to embryonic lethality, displaying abnormal vessel branching, massive hemorrhage and EC apoptosis, indicating a redundant and crucial role between ETS1 and ETS2 in developmental angiogenesis (25,26). It has been shown that expression of ETS1 in tumor cells promotes vascular mimicry by induction of receptor for vascular endothelial growth factor (27). Vascular abnormality is associated with a distinct gene signature in ECs (10,28). ETS1 could control the expression of several genes driving vascular abnormality, including MCAM (29), ANGPT2 (30), SOX4 (31), VEGFA/VEGFR2 (25,32), ITGA1 (33), NOTCH4 (34,35). However, the direct effects of ETS1 on function of tumor ECs remain poorly defined. Here, we showed that ETS1 was upregulated in GBM ECs compared to ECs from non-malignant control brains. Knockdown of Ets1 in mouse brain endothelial cells inhibited cell migration and proliferation, and suppressed expression of genes associated with vascular abnormality in GBM. ETS1 upregulation in tumor ECs was dependent on TGFβ signaling, and targeting TGFβ signaling by inhibitor decreased tumor angiogenesis and vascular abnormality in CT-2A glioma model.

## Materials and Methods

### Bioinformatics Analysis of ETS1 Expression in ECs

Single cell RNA sequencing datasets of ECs from GBM and paired non-malignant control brain tissue were downloaded from the Gene Expression Omnibus (GEO) database (GSE162631). Information for cells and samples were obtained in the previous study (4). Only ECs from peripheral endothelial cell type I (Pe1), tumor core endothelial cell type I (Co1) and tumor core endothelial cell type II (Co2), which were represented in four patients, in the original study were selected for downstream analysis. 416 ECs from Pe1 cluster were considered as non-malignant brain endothelial cells. 634 ECs from Co1 and Co2 clusters were considered as tumor endothelial cells.

The expression of ETS1 in different human GBM anatomic regions, including leading edge region, infiltrating tumor region, cellular tumor core region, pseudopalisading necrosis region and microvascular proliferation region, was obtained from Ivy GAP database (http://glioblastoma.alleninstitute.org).

### Patient Information and Ethical Considerations

Ethical permission for using patient samples was granted by the Ethics Committee of Hunan University of Medicine (HUM-HE-2019-015). Glioblastoma samples were collected retrospectively at the First Affiliated Hospital of Hunan University of Medicine. For sample details, see Supplementary Table S1.

### Cell Culture and siRNA Transfection

Murine brain endothelial cell (bEND.3) was purchased from Chinese Academy of Sciences Cell Bank. The CT-2A mouse glioma cell line was a gift from Dr. L. Zhang, Shaanxi Normal University. bEND.3 and CT-2A cells were cultured in Dulbecco’s modified Eagle’s medium (DMEM) (ThermoFisher, 10566016) supplemented with 10% fetal calf serum (FCS) (ThermoFisher, 10091155).

For Ets1 knockdown, bEND.3 cells were seeded and incubated with control siRNA or siRNA to Ets1 at concentration of 10 nmol/L using siRNA-mate (GenePharma) according to the manufacturer’s manual. The sequence of siRNA for Ets1 targeting was listed as following: control siRNA (siNT), sense: UUC​UCC​GAA​CGU​GUC​ACG​UTT, anti-sense: ACG​UGA​CAC​GUU​CGG​AGA​ATT; siEts1-331, sense: GGA​CAA​GCC​UGU​CAU​UCC​UTT, anti-sense: AGG​AAU​GAC​AGG​CUU​GUC​CTT; siEts1-946, sense: GGA​AUU​ACU​CAC​UGA​UAA​GTT, anti-sense: CUU​AUC​AGU​GAG​UAA​UUC​CTT. Experiments were performed on day 2–3 after siRNA transfection. Ets1/Ets1 knockdown efficiency was determined by qPCR and western blot 48 h after transfection.

### Cell Proliferation Assay

Cell proliferations were determined using Cell Counting Kit-8 (CCK8, Beyotime Biotechnology). In brief, 200 μl of cell suspension (2.5 × 10^4^ cells/ml) was placed into each well of Primaria 96-well plates (BD Biosciences), performing triplicates for each time-point. Three wells with media alone were used for determination of background in each experiment. On day 1 and day 7, 20 μl CCK8 was added to each well, the plate was incubated at 37°C for 2 more hours. The number of living cells were indicated by the absorbance at 450 nm detected by INFINITE M NANO absorbance plate reader (TECAN). The cell density on day 7 was normalized to that on day 1 shown as the proliferation index. The experiment was repeated four times.

### *In Vitro* Stimulation of Endothelial Cells

To determine the effect of VEGFA, TNFα or TGFβ2 on *Ets1* expression in bEND.3 cells, the bEND.3 cells were seeded on the 12-well plates (8 × 10^4^ cells/well). At the second day, the cells were starved in DMEM with 1% FCS for overnight, followed by VEGFA (50 ng/ml; Peprotech), TNFα (2 ng/ml; Peprotech) or TGFβ2 (10 ng/ml, Peprotech) stimulations for 48 h. The experiment was repeated three times with 3 samples per group in each experiment. To determine whether *Ets1* was up-regulated by tumors through TGFβ signaling *in vitro*, bEND.3 cell were seeded on the 12-well plates (8 × 10^4^ cells/well). After starvation in DMEM with 1% FCS for overnight, the bEND.3 cells were cultured with conditioned medium from CT-2A glioma cells for 48 h. The conditioned medium was collected from confluent CT-2A glioma cells culture, and then filtered through 0.2 μm Nalgene Syringe filter (ThermoFisher, 720-1320), followed by pre-treatment with 50 μg/ml anti-TGFβ neutralizing antibody (ThermoFisher, 16-9243-85) or control antibody (R&D system, MAB002) overnight (12 h), before incubation with bEND.3 cells. The experiment was repeated three times with 3 samples per group in each experiment.

### Scratch Wound Migration Assay

A scratch wound was applied on confluent cell monolayers using a 200 ul tip. Pictures were taken at 0 (T0) and 24 h (T24) post-scratching using a Primovert iLED microscope (Zeiss, Germany). Migration was measured with the fiji/ImageJ software and is expressed as % wound closure (gap area at T0 minus gap area at T24 in % of gap area at T0). The experiment was repeated three times with 4 samples per group in each experiment.

### Transwell Migration Assay

Transwell inserts with 8 µm pore size (CLS3422-48 EA, Corning) for 24-well plates were used and 5 × 10^4^ cell were seeded in the upper chamber in medium without fetal bovine serum (Biological Industries). Regular culture medium containing fetal bovine serum was added to the lower chamber. After 24 h, cells were fixed with 4% formaldehyde, and permeabilized with methanol. Non-migrated cells were removed from the upper surface of the membrane and the membrane was cut off and stained with Hoechst 33258 (Sigma-Aldrich). To quantify the cells that had migrated through the membrane, pictures were taken at four different fields using an Axio Imager upright microscope (Zeiss, Germany). The experiment was repeated three times with 8 samples per group in each experiment.

### Quantitative Polymerase Chain Reaction

Total RNA was extracted with RNeasy Mini Kit (Qiagen, 74104). cDNA from total RNA was synthesized using random hexamer primers and SuperScript III reverse transcriptase (ThermoFisher, 18080093) according to the manufacturer’s instructions. qPCR was performed on Thermal Cycler iQ5 multicolor Real-Time PCR detection system (Bio-Rad) using TB Green Premix Ex TaqTMII (Takara, RR820A) with 0.25 μM reverse and forward primer per well. Gene expression was normalized to the house-keeping gene hypoxanthine guanine phosphoribosyl transferase (*Hprt*) according to the following formula: relative expression of gene X = 2^-(CT^ ^*Hprt*− CT gene X)^. The sequences of primers for qPCR were listed as following: *Angpt2*, forward: CCT​CGA​CTA​CGA​CGA​CTC​AGT, reverse: TCT​GCA​CCA​CAT​TCT​GTT​GGA; *Mcam*, forward: CCC​AAA​CTG​GTG​TGC​GTC​TT, reverse: GGA​AAA​TCA​GTA​TCT​GCC​TCT​CC; Sox4: forward: CGG​CTG​CAT​CGT​TCT​CTC​C, reverse: CGC​TTC​ACT​TTC​TTG​TCG​GC; *Vegfa*, forward: CTG​CCG​TCC​GAT​TGA​GAC​C, reverse: CCC​CTC​CTT​GTA​CCA​CTG​TC; *Kdr*, forward: TTT​GGC​AAA​TAC​AAC​CCT​TCA​GA, reverse: GCA​GAA​GAT​ACT​GTC​ACC​ACC; *Itga1*, forward: CCT​TCC​CTC​GGA​TGT​GAG​TCA, reverse: AAG​TTC​TCC​CCG​TAT​GGT​AAG​A; *Notch4*, forward: CTC​TTG​CCA​CTC​AAT​TTC​CCT, reverse: TTG​CAG​AGT​TGG​GTA​TCC​CTG.

### Western Blot

Cells were lysed in Pierce LDS sample buffer (ThermoFisher, 84788) and protein concentration was determined using BCA protein assay kit (BCA, Beyotime Biotechnology). 10 μg of protein was loaded on the gel. Samples were separated on NuPAGE 4–12% Bis-Tris gels (ThermoFisher, NP0335BOX) using MOPS SDS running buffer (ThermoFisher, NP0050), and then transferred to a Hybond-C Extra filter (GE Healthcare). Membranes were blocked with 5% milk in tris-buffered saline plus 0.01% Tween, and incubated with primary antibodies (anti-Ets1 antibody: abcam, ab220361; anti-β-actin antibody: abcam, ab8227) diluted in blocking solution overnight at 4°C. Then, membranes were incubated with horseradish peroxidase (HRP)-conjugated secondary antibodies (abcam, ab205718), and detected using Pierce ECL plus substrate (ThermoFisher, 32134).

### Orthotopic CT-2A Glioma Model and Study Approval

Mouse studies were approved by the Animal Experiment Ethical Committee of Hunan University of Medicine (HUM-AE-2018-113). Six-week-old C57BL/6 mice were purchased from Vitalriver. During injection, mice were anesthetized with 2.5% isoflurane. CT-2A cells (5 × 10^4^) in 2 μl Dulbecco’s phosphate-buffered saline (DPBS) were injected into subventrical zone (coordinates: 1 mm anterior to bregma, 1.5 mm from the mid-line, and 2.7 mm below the cranial surface) using the Hamilton microtiter syringe. Pre-warmed pads were employed for mice until fully recovered. Tumor-bearing mice were administered daily with galunisertib (HY-13226, MedChemExpress) by oral gavage (150 mg/kg of galunisertib in 0.5% methylcellulose/Tween 80) starting from 7 days after tumor inoculation. 10 days after first treatment, tumor-bearing mice were sacrificed, and brains were collected for further analysis. The experiment was repeated two times with at least 10 mice per group in each experiment.

### Immunohistochemistry Analysis and Quantification

Immunohistochemistry (IHC) staining of ETS1, CD31 was performed on 6 μm paraffin sections of mouse brain or human tumor. Sections were deparaffinized and dehydrated prior to antigen retrieval followed by blocking with 3% bovine serum albumin (BSA) (Sigma-Aldrich, A7906) in phosphate-buffered saline (PBS). Then the sections were incubated with primary antibodies against Ets1 (Abcam, ab220361), CD31 (Dianova, DIA-310) followed by incubation with biotinylated secondary antibody (Vector Laboratories, BA-1000, anti-rabbit IgG; BA-9400, anti-rat IgG) and streptavidin conjugated to peroxidase (Vector Laboratories, SA-5014). The staining was detected with DAB substrate (Vector Laboratories, SK-4100) according to manufacturer’s instruction. The hematoxylin counterstaining was used to visualize nuclei. The images were acquired using NIS software (Nikon). The Ets1 staining was semi-quantified according to the fraction of positively stained vessels on a scale from 0 to 2 (no vessel stained, minority vessel stained, majority vessel stained). The vascular areas were quantified according to the area stained positive for CD31 using Image J software. The data is presented as CD31 positive area in a given area (μm^2^ mm^−2^).

### Stereological Quantification of Vascular Space

Tumor vascular space, indicated by the mean diameters of the vessels, were analyzed on CD31 immunohistochemical stained paraffin sections using eyepiece grid as previously described (36,37). In brief, the eyepiece grid with 10 × 10 squares (0.25 mm × 0.25 mm) was placed at tumor area. The number of vessels (Q_ves) and the number of test points hitting vessels (P_ves) were counted in the counting frame. 10–25 frames were quantified from each tumor depending on the tumor size. The mean vascular diameters were calculated based on the following formulation (37): $d\left(\mathrm{mean}\;section\;diameter\;of\;vessels;mm\right)=2\times \sqrt{\frac{\sum P\_\mathrm{ves}}{\sum Q\_\mathrm{ves}}\times \frac{A\left(\mathrm{frame}\right)}{2\pi \times P\left(\mathrm{pcg}\right)}}$ A (frame) (area of one counting frame) = 0.0625 mm^2^; P (pcg) (number of test **p**oints in one **p**oint-**c**ounting **g**rid) = 121.10 individual tumors were analyzed per group.

### Statistical Analysis

Statistical analysis was performed using GraphPad Prism software and R software. The Mann-Whitney test or t test was performed to determine statistically significant differences in the experiments with two groups. One-way ANOVA with Tukey’s multiple comparisons test was performed to determine statistically significant differences in the experiments with more than two groups. All statistical tests were two-sided.

## Results

### ETS1 is Upregulated in GBM ECs

**FIGURE 1:**
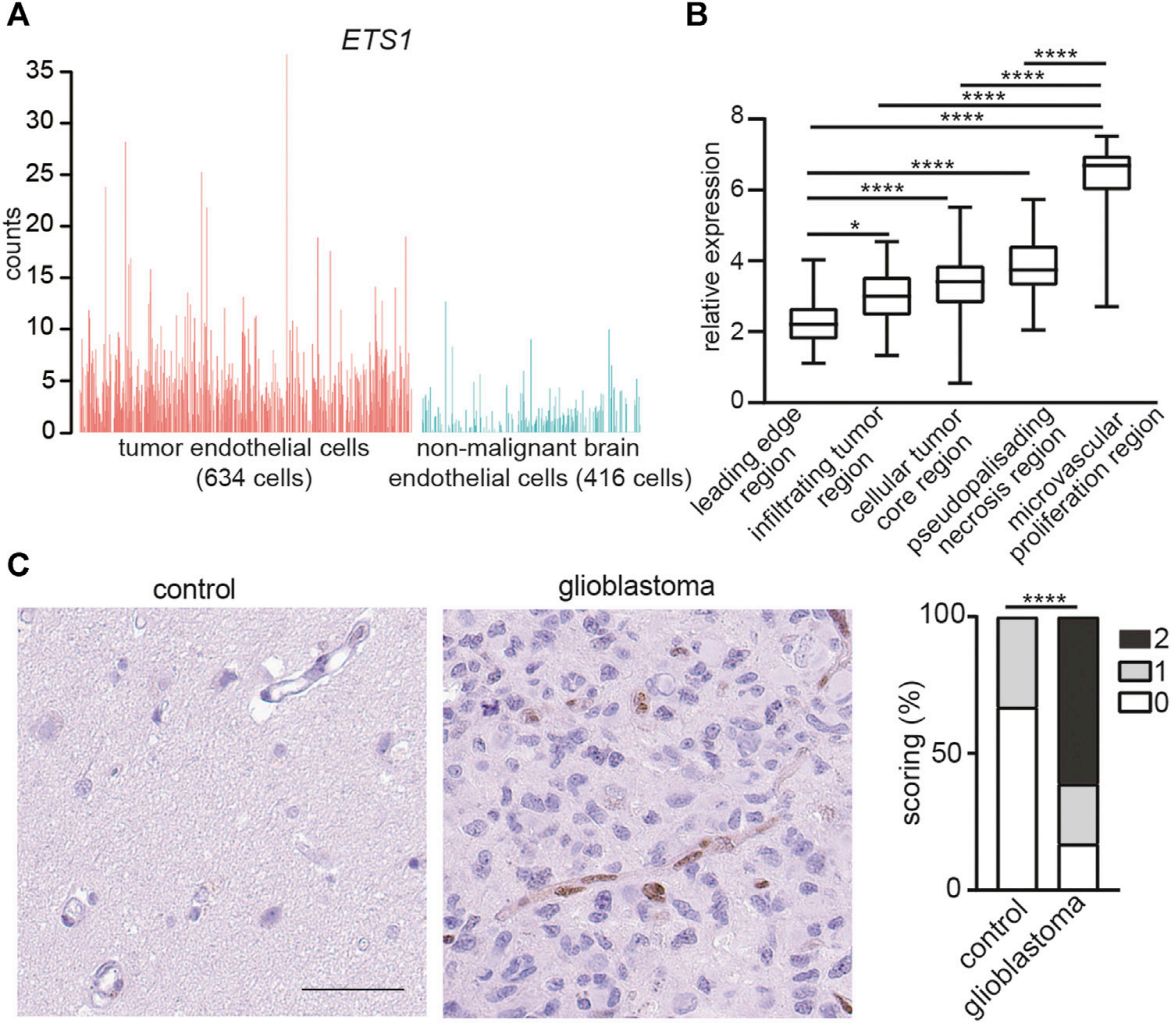
ETS1 is upregulated in tumor endothelial cells in human glioblastoma. **(A)** Bar plots showing *ETS1* expression in tumor endothelial cells (red) and non-malignant brain endothelial cells (blue) (GSE162631). **(B)** Expression of *ETS1* in Ivy GAP RNA-seq of distinct GBM anatomic structures (One-way ANOVA with Tukey’s multiple comparisons test; *p < 0.05, ****p < 0.0001). **(C)** Immunohistochemical staining and quantification of ETS1 in human GBM and paired non-malignant brain tissue (bar: 50 μm) (*n* = 18, Mann-Whitney test, ****p < 0.0001).

To investigate the expression of *ETS1* in GBM ECs, we reanalyzed a recently published dataset of single cell RNA-seq (scRNA-seq) of ECs from tumors and paired non-malignant brain tissue in 4 GBM patients (4). *ETS1* was upregulated in ECs from tumor compared to non-malignant brain ECs (Student’s t test; *p* < 0.0001) (Figure 1A). The expression of *ETS1* in distinct anatomical locations of 34 GBM samples was analyzed by using Ivy GAP database (http://glioblastoma.alleninstitute.org/) (38), which documented transcriptome from microdissected human GBM anatomic regions, including leading edge, infiltrating tumor region, cellular tumor core region, microvascular proliferation region and pseudopalisading necrosis region. In accordance with upregulation of ETS1 in tumor ECs, *ETS1* expression was higher in microvascular proliferation region where tumor ECs were enriched due to active angiogenesis (Figure 1B). Upregulation of ETS1 in tumor vasculature was further confirmed in protein level by immunohistochemical staining of our in-house samples including 18 GBM tumors and paired control brain tissue (Figure 1C). Taken together, these results indicate that ETS1 is upregulated in GBM ECs.

### TGFβ Increases *Ets1* Expression in Brain ECs

**FIGURE 2:**
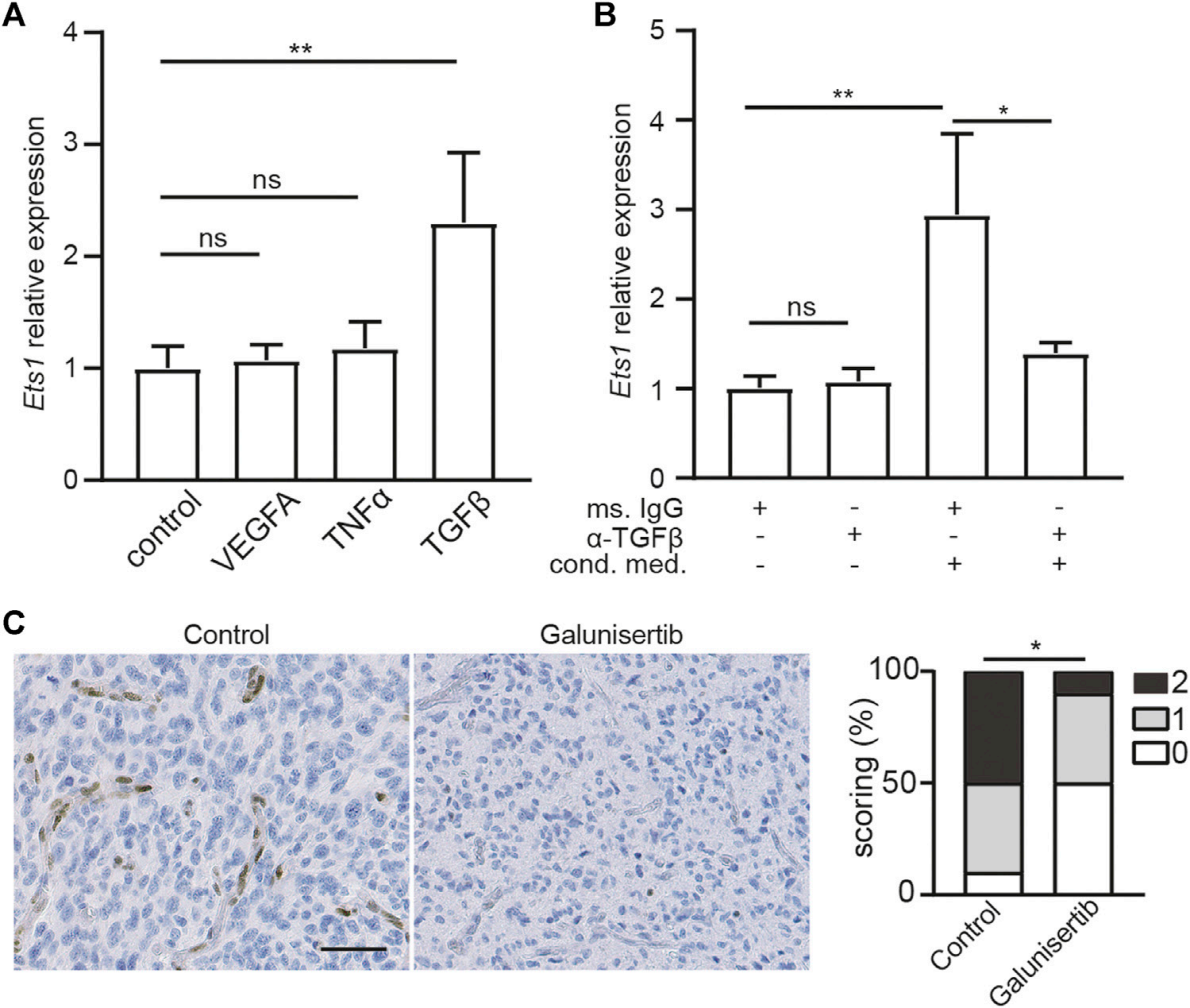
Ets1 upregulation in glioblastoma endothelial cells depend on TGFβ signaling. **(A)** RNA levels of *Ets1* in bEND.3 cells treated with VEGFA, TNFα or TGFβ (mean + SD of four independent experiments; **p < 0.01; ANOVA with Tukey’s multiple comparisons test). **(B)** RNA levels of *Ets1* in bEND.3 cells treated with conditioned medium from CT-2A glioma cells with TGFβ neutralizing antibody or control antibody (mean + SD of four independent experiments; **p < 0.01; One-way ANOVA with Tukey’s multiple comparisons test). **(C)** Immunohistochemical staining and quantification of Ets1 in CT-2A glioma tumor-bearing mice treated with/without galunisertib (bar: 50 μm) (*n* = 10/group, Mann-Whitney test, *p < 0.05).

Regulation of *ETS1* expression in brain ECs is still unknown. To uncover the signal pathway mediating *ETS1* upregulation, we analyzed the expression of *Ets1* in bEND.3 cells upon stimulation of VEGFA, TNFα and TGFβ2, which can increase *ETS1*/*Ets1* expression in human umbilical vein endothelial cells (HUVECs) or renal cells (39–41). Neither TNFα nor VEGFA could increase *ETS1* expression in bEND.3 cells (Figure 2A). Notably, TGFβ treatment could upregulate *ETS1* expression (Figure 2A), indicating a key role of TGFβ signaling on mediating *ETS1* induction in brain ECs.

### Ets1 Upregulation in GBM ECs is Dependent on TGFβ Signaling

To determine whether ETS1 is upregulated in GBM ECs via TGFβ-dependent manner, bEND.3 cells were stimulated with CT-2A glioma cells conditioned medium with neutralizing antibody against TGFβ or control antibody, after which the expression of *Ets1* was analyzed by qPCR. We found that conditioned medium from GBM tumor cells was sufficient to upregulate *Ets1* expression in bEND.3 cells (Figure 2B), suggesting a direct effect of tumor cell on Ets1 expression in ECs. Notably, neutralizing TGFβ antibody treatment attenuated tumor conditioned medium induced upregulation of *ETS1* expression in bEND.3 (Figure 2B). To evaluate the role of TGFβ signaling on Ets1 expression in tumor ECs *in vivo*, we employed CT-2A syngeneic orthotopic glioblastoma model. CT-2A tumor-bearing mice were treated with galunisertib, a potent TGFβ receptor kinase inhibitor (42,43). In accordance with *in vitro* findings, galunisertib treatment decreased Ets1 level in tumor vessels *in vivo* (Figure 2C). These results indicate that Ets1 upregulation in GBM ECs is dependent on TGFβ signaling.

### Knockdown of Ets1 Inhibits Brain ECs Migration and Proliferation *In Vitro*

**FIGURE 3:**
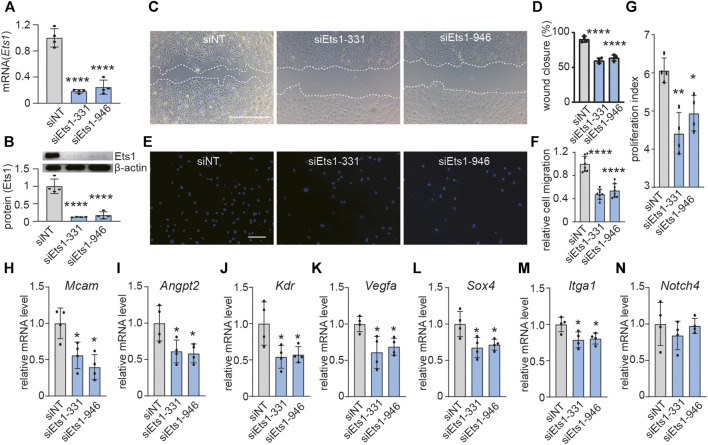
Ets1 knockdown inhibits expression of genes associated with vascular abnormality. **(A)** *Ets1* mRNA expression determined by qPCR in bEND.3 cells transfected with siNT (control siRNA) or siEts1 (mean + SD of four independent experiments). **(B)** Ets1 protein expression by western blot in bEND.3 cells trandfected with siNT or siEts1 **(Top)**. Western blot quantification was performed with software Image J **(bottom)** (mean + SD of four independent experiments). **(C, D)** Micrographs **(C)** and quantification **(D)** of control or Ets1 silenced bEND.3 cell migration in scratch wound assays (bar: 200 μm) (*n* = 4/group). **(E, F)** Micrographs **(E)** and quantification **(F)** of control or Ets1 silenced bEend.3 cell migration in transwell migration assay (bar: 100 μm) (*n* = 8/group). **(G)** Quantification of control or Ets1 silenced bEend.3 cells in proliferation assay. **(H–N)** Quantification of mRNA expression of vascular abnormality associated genes including *Mcam* **(H)**, *Angpt2* **(I)**, *Kdr* **(J)**, *Vegfa* **(K)**, *Sox4* **(L)**, *Itga1* **(M)** and *Notch4* **(N)** in control or Ets1 silenced cells (*n* = 4/group). Data represent the mean ± SD, One-way ANOVA with Tukey’s test, *p < 0.05, **p < 0.01, ****p < 0.0001.

Active angiogenesis is a key feature of GBM vessels (28). To investigate the role of Ets1 on angiogenesis, we determined whether Ets1 knockdown affects the migratory capacity and proliferation of bEND.3 cells. We used RNA interference to knockdown the expression of Ets1 in bEND.3 *in vitro*. Transfection of bEND.3 cells with siRNA to Ets1 resulted in an efficient downregulation of Ets1 in both RNA and protein levels (Figures 3A,B). Ets1 knockdown led to a significant reduction of bEND.3 cell migration compared to control cells (Figures 3C,D). These results were further supported by transwell migration assay showing that the migration of bEND.3 cells through membrane was inhibited by Ets1 knockdown (Figures 3E,F). In addition, knockdown of Ets1 inhibited proliferation of bEND.3 cells (Figure 3G). Taken together, these results suggest that Ets1 may regulate brain EC migration and proliferation.

### ETS1 Regulates Expression of Genes Associated With Vascular Abnormality in Brain ECs

To investigate the role of ETS1 on expression of genes associated with vascular abnormality in brain ECs. qPCR analysis revealed that Ets1 knockdown in brain ECs suppressed the expression of 6 out of 7 selected vascular abnormality associated genes (Figures 3H–N), including *Mcam*, *Angpt2*, *Kdr*, *Vegfa*, *Sox4* and *Itga1*, suggesting a potential role of Ets1 on vascular abnormality.

### TGFβ Inhibition Decreases Angiogenesis and Vascular Abnormality in CT-2A Glioma

**FIGURE 4:**
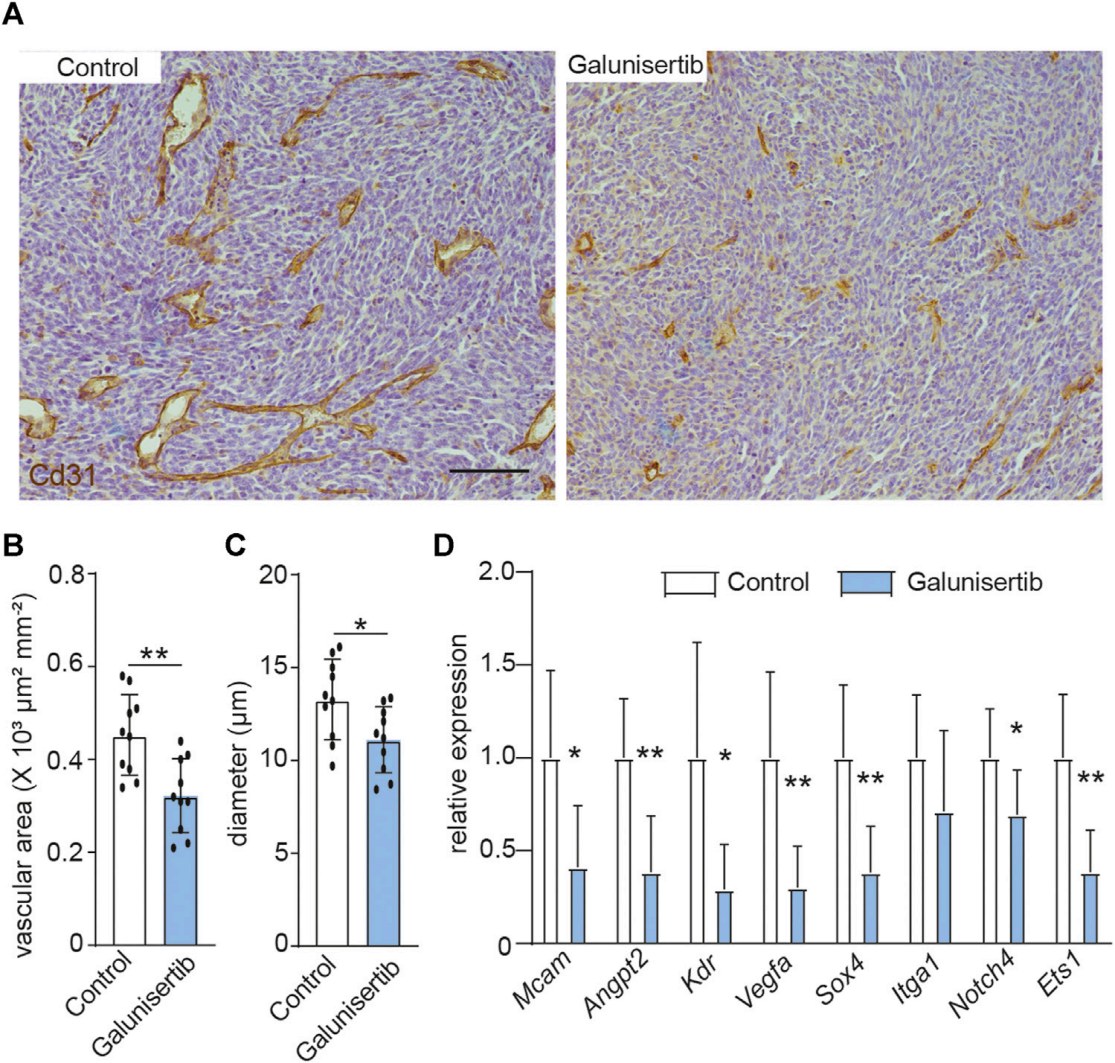
TGFβ inhibition decreases angiogenesis and vascular abnormality in CT-2A glioma. **(A)** Immunohistochemical staining and quantification of CD31 in CT-2A glioma tumor-bearing mice treated with or without galunisertib (bar: 100 μm). **(B, C)** Stereological quantification of vessel area **(B)** and mean vessel diameters **(C)** in CT-2A glioma tumor-bearing mice treated with or without galunisertib (bar: 100 μm) (*n* = 10/group, unpaired t test, *p < 0.05). **(D)** Quantification of mRNA expression of vascular abnormality associated gene including *Mcam*, *Angpt2*, *Kdr*, *Vegfa*, *Sox4*, *Itga1* and *Notch4* in CT-2A glioma tumor-bearing mice treated with or without galunisertib (*n* = 7/group, unpaired t test, *p < 0.05, **p < 0.01).

We next set out whether TGFβ signaling, which is the upstream of ETS1 signaling (Figure 2C), affects the tumor angiogenesis and vascular abnormality. Quantification of vessel density, based on CD31 positive staining area, revealed decreased vascular area upon TGFβ inhibition (4A-4B). In addition, the space occupied by the blood vessel in CT-2A tumors, indicated by mean vascular diameter, was reduced in the tumors upon galunisertib treatment according to stereological quantification (36), (Figures 4A,C), indicative of an effect of TGFβ inhibition on angiogenesis and vascular normalization. We additionally analyzed the expression of a panel genes (*Angpt2*, *Sox4*, *Vegfa*, *Kdr*, *Itga1*, *Mcam*, *Notch4* and *Ets1*) associated with vascular abnormality in ECs using RNA extracted from total glioma tumor tissues from control and treated mice. We found that 7 out of 8 selected genes including *Angpt2*, *Sox4*, *Vegfa*, *Kdr*, *Mcam*, *Notch4* and *Ets1* were downregulated in tumors upon galunisertib treatment (Figure 4D). Taken together, these results indicate that targeting TGFβ signaling, the upstream of ETS1, could suppress tumor angiogenesis and downregulate vascular abnormality associated genes.

## Discussion

Ets1 is a key transcription factor regulating EC differentiation and function (44). The role of Ets1 on developmental angiogenesis had been well characterized in both mice and zebrafish (25,26). However, the role of Ets1 on tumor angiogenesis remains largely unknown. Here, by analysis of scRNA-seq dataset of ECs from tumors and paired non-malignant brain tissue together with immunostaining of patient samples, we found that ETS1 is upregulated in ECs in GBM.

ETS1 can act as both pro- and anti-angiogenic factor. The pro-angiogenic role ETS1 has been convincingly demonstrated in several studies through both *in vivo* and vitro models (19,44–47). A recent study uncovered the key role of ETS1 on VEGF mediating broad transcription amplification (48), providing molecular mechanisms linking ETS1 with angiogenesis. ETS1 chromatin occupancy and acetylation are enhanced upon VEGF activation, leading to recruit machinery components to promote RNA polymerase pause release (48). In contrast, ETS1 expression is induced in ECs upon Fzd5 loss, and acts as an anti-angiogenic factor suppressing angiogenesis by transcription activation of vascular destabilizing factors including ANGPT2 and FLT1 (30). The contradictory effect of ETS1 on angiogenesis may depend on the cues in the microenvironment, such as distinct levels of VEGF (49). In the present study, ETS1 knockdown attenuated GBM cells induced EC migration and proliferation, indicative of a pro-angiognic role of ETS1 in GBM.

Ets1 knockdown in brain ECs could suppress a panel of genes associated with vascular abnormality, including *Vegfa*, *Kdr*, *Angpt2*, *Sox4* and *Mcam*. VEGFA/KDR is the key pathway triggering microvascular proliferation and vascular abnormality in GBM (28). Angpt2 is an angiogenic factor which mediates resistance to bevacizumab in GBM (50). SOX4 and Mcam are upregulated in glioblastoma vessels and could promote tumor angiogenesis (28,51–53).

By neutralizing TGFβ *in vitro* and inhibition of TGFβ signaling *in vivo*, we demonstrate that ETS1 upregulation in GBM ECs is dependent on TGFβ signaling. TGFβ signaling is dysregulated in glioblastoma, and this aberrant signaling contributes to tumor progression through multiple biological processes, including promoting tumor cell proliferation, enhancing tumor invasion, suppressing anti-tumor immune response, maintaining self-renewal capacity of glioma stem cells and activating angiogenesis [reviewed in Ref. (54)]. VEGF signaling inhibition with VEGF antibody B20-4.1.1 leads to an improvement of survival in several murine glioblastoma models, accompanied with reduced tumor volume and blood vessel density (55). TGFβ signaling inhibition with galunisertib results in a reduction of phosphorylated SMAD2 in tumor cells, but not a survival improvement (55). Notably, VEGF and TGFβ signaling co-inhibition is superior to either treatment alone in GL261 model, suggesting a synergistic anti-tumor effect (55). Our study further uncovered TGFβ/ETS1 axis as a novel pathway regulating glioblastoma angiogenesis and vascular abnormality.

A potent small inhibitor of ETS1 (YK-4-270) was identified recently, which reduced neovascular tufts in retinal vessels in an oxygen-induced retinopathy model (56). Our results support further research to investigate the therapeutic potential of this inhibitor as vascular targeting drug in GBM treatment.

Taken together, our data uncover a key role of ETS1 on microvascular proliferation and abnormality in GBM.

## Data Availability

Publicly available datasets were analyzed in this study. This data can be found here: GSE162631.
